# Improving data transparency in clinical trials using blockchain smart contracts

**DOI:** 10.12688/f1000research.9756.1

**Published:** 2016-10-20

**Authors:** Timothy Nugent, David Upton, Mihai Cimpoesu

**Affiliations:** 1 Corporate Research and Development, Thomson Reuters, London, UK; 2 Applied Innovation, Thomson Reuters, London, UK

**Keywords:** Clinical Trials, Missing Data, Blockchain, Ethereum, Smart Contract

## Abstract

The scientific credibility of findings from clinical trials can be undermined by a range of problems including missing data, endpoint switching, data dredging, and selective publication. Together, these issues have contributed to systematically distorted perceptions regarding the benefits and risks of treatments. While these issues have been well documented and widely discussed within the profession, legislative intervention has seen limited success. Recently, a method was described for using a blockchain to prove the existence of documents describing pre-specified endpoints in clinical trials. Here, we extend the idea by using smart contracts - code, and data, that resides at a specific address in a blockchain, and whose execution is cryptographically validated by the network - to demonstrate how trust in clinical trials can be enforced and data manipulation eliminated. We show that blockchain smart contracts provide a novel technological solution to the data manipulation problem, by acting as trusted administrators and providing an immutable record of trial history.

## Introduction

Data from clinical trials is routinely withheld from researchers, doctors, and patients, leading to a lack of trust in the process and highlighting the need for greater transparency ^1^. While there have been efforts by the World Health Organization (WHO) requiring all trials to make their methods and results available ^2^, a view supported by the UK Medicines and Healthcare products Regulatory Agency (MHRA), it remains to be seen how effectively such statements can be enforced. For example, while United States Food and Drug Administration (FDA) regulations require that methods and results of all clinical trials be made available, a recent study suggests that more than half of trials have failed to do so ^3^ Clearly, legislation alone will not solve these problems. Technological solutions such as the use of blockchains for record management may therefore provide an alternative strategy with which to address these challenges.

A blockchain serves as a distributed database which maintains a continuously growing list of transactional records organised into blocks, using consensus algorithms allowing untrusted parties to agree on a common state while ensuring tamper resistance. Valid transactions stored in a blockchain are digitally signed and timestamped by their sender, providing cryptographically irrefutable evidence of both the provenance and existence of a record at a given point in time. These qualities were recently leveraged by Carlisle and Irving and Holden to address endpoint switching in clinical trials [10] ^4^. Using the public Bitcoin blockchain ^5^ - perhaps the best known example of a blockchain - they generated a hash of a study protocol document, and used this as a public address to which they sent a transaction. This process serves as a “proof-of-existence” - verification that the document exists at the timestamp indicated by the transaction. Since Nakamoto’s seminal Bitcoin paper, blockchains have moved into the 2.0 era with the advent of smart contracts - code, and data, that resides at a specific address in a blockchain, and whose execution is cryptographically validated by the network. Here, we introduce a system built using smart contracts which addresses a number of the data manipulation issues common to clinical trials. We show that smart contracts can act as trusted administrators, able to improve the transparency of data reporting in clinical trials, by immutably capturing all aspects of data that might be subject to manipulation including trial registration, protocol, subject registration, and clinical measurements.

## Methods

**Figure 1.**
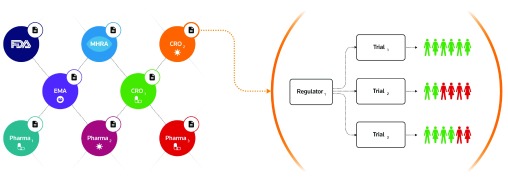
A private blockchain network consisting of regulators, pharma and contract research organisations. The system is composed of a hierarchical arrangement of two core types of smart contract - regulator contracts and trial contracts - with subjects and their associated clinical measurements appended to a container within the trial contract. The logic within the trial contract effectively enforces aspects of the trial protocol, ensuring that neither subjects nor measurements are appended outside of the predetermined trial timelines, while the tamper resistant characteristics of the blockchain prevent data manipulation.

We propose a private, permissioned Ethereum blockchain network maintained by regulators (e.g. MHRA, FDA), pharma and contract research organisations (CROs), to be used in parallel with traditional clinical data management systems (CDMS), framing the process as a transactional inter-organisational record keeping model between untrusted participants ( Figure 1). Ethereum is a blockchain protocol that features smart contract functionality, and has been described as a next-generation cryptocurrency and decentralised application platform ^6, 7^. Rather than validating just the balances and transfer of digital tokens, smart contracts allow the state of arbitrary data and logic to be agreed on by the network using the same cryptographic principles. A hierarchical arrangement of two core types of smart contract is required:

(i) A regulator contract, holding a data structure containing clinical trial authorisation (CTA) details. This contract is owned and updated by regulators based on off-chain licensing agreements, and includes a container used to store trial contracts.

(ii) A trial contract, deployed by CROs using a function within the regulator contract, dependent on permissioning logic determined using the CTA data structure. Contains a data structure used to store the trial protocol, using IPFS ^8^ or Ethereum’s native Swarm protocol where large file storage is required, with permissioning logic requiring protocol deposition and endpoint definition prior to the storage of subjects within a container.

Subjects are added by CROs using a function within the trial contract, with permissioning logic restricting the calling of this function outside of the recruitment period defined in the protocol. The subject data structure contains anonymised subject information, consent documentation, and a container allowing storage of successive clinical measurements. Individual measurements are recorded, with full timestamping, in a format such as string-encoded JavaScript Object Notation (JSON), providing a flexible schema that can be adapted to any study type. Should data privacy be required, strings can be encrypted using public key encryption, with regulators holding a distinct private key for each trial contract, or using more elaborate techniques such as zero-knowledge proofs and homomorphic encryption as they become available.

Source code written in JavaScript and the Solidity smart contract programming language is provided under Data and software availability, allowing contracts to be implemented, and data to be written to and read from the blockchain. The scripts perform the following steps:

- Start JavaScript implementations of Ethereum and IPFS nodes, each connecting to local private networks.
- Deploy a regulator contract. A trial proposal, including protocol documentation, is subsequently submitted to this contract by a CRO, with the documentation being stored using IPFS.
- If the proposal is accepted by the regulator, a trial contract is created. This contract is owned and administered by the CRO.
- Subjects are appended to the trial contract up until the trial start data. Synthetic data is then appended for each of the subjects, up until the trial end date.
- Finally, a script is provided to read all the data from the blockchain, providing a summary of each trial, and details of each subject and data points that have been added, with full timestamping.

Data and full source code required to repeat the experiment See README.txt for a description. Click here for additional data file. Copyright: © 2016 Nugent T et al. 2016 Data associated with the article are available under the terms of the Creative Commons Zero "No rights reserved" data waiver (CC0 1.0 Public domain dedication).

## Results

Contracts were deployed onto a private Ethereum blockchain and used to record synthetic data representing clinical trials of Tamiflu, an influenza drug stockpiled by the British government at a cost of £424m despite 60% of trial data remaining unpublished at the time the decision was taken ^9^, totalling thousands of individual transactions. Ethereum’s block time is significantly faster than the Bitcoin blockchain, with transactions used to deploy contracts or update data taking an average of 14 seconds to be accepted by the network, although confirmation of 12 blocks is recommended to ensure finality. With the Ethereum roadmap anticipating the processing of 10,000 transactions per block by release 2.0, the network should scale well for the task in hand. At all points during the test, we were able to query the number of trials underway, the number of subjects recruited to each one, the address of the transaction sender (resolvable to a CRO) and the timestamp at which the transaction was processed. Due to the append-only nature of blockchains, we were also able to query the state of the data at any historic block.

## Conclusions

Here, we have demonstrated that smart contracts running on the Ethereum blockchain can be used to improve the transparency of data management in clinical trials. We have shown that the cryptographic guarantees that modern protocols provide can go beyond “proof-of-existence”, and be used for complex clinical trial data management that prevents all forms of manipulation due to the tamper resistant characteristics of blockchains. Systems built using smart contracts should help to increase trust in the data they hold and the credibility of trials findings, allowing medical professionals to make better-informed decisions that have the potential to reduce both patient risk and the financial strain placed on health services that data manipulation issues contribute to.

## Data and software availability

F1000Research: Dataset 1. Data and full source code required to repeat the experiment., 10.5256/f1000research.9756.d138647 ^11^
