# Supplementary material for: Improving data transparency in clinical trials using blockchain smart contracts
Source: F1000Res. 2016 Oct 20;5:2541. [Version 1] doi: 10.12688/f1000research.9756.1 (PMC5357027; doi:10.12688/f1000research.9756.1)
Supplement: Data and full source code required to repeat the experiment — See README.txt for a description. [file f1000research-5-10518-s0000.tgz › d9f7cbf0-8cae-49d3-b2f6-049a10fc4b81_bffe73a6-4133-4912-ba15-e474df461a16_true-clinical_(2).zip!/true-clinical/data/TrialProtocol.pdf]

Purpose

The purpose of this study is to learn how to treat influenza in children less than 2 years of age. Tamiflu®, the drug being studied, is approved for treatment of children 1 year of age and older with influenza. Researchers want to learn more about the activity of Tamiflu® in the body to determine a dose of that is safe, well-tolerated, and effective in young children with influenza. Children less than 24 months of age with confirmed influenza will receive Tamiflu® 2 times a day for 5 days. Older participants will be enrolled first and younger children will be enrolled after the safety data is reviewed for older participants. Study procedures include blood samples, swabs from inside the nose, and body and nervous system evaluations. Participants may be involved in study related procedures for up to 37 days.

Condition	Intervention	Phase
Influenza	Drug: oseltamivir (Tamiflu®)	Phase 1<br>Phase 2

Study Type: Interventional

Study Design: Allocation: Non-Randomized
Endpoint Classification: Pharmacokinetics/Dynamics Study
Intervention Model: Parallel Assignment
Masking: Open Label
Primary Purpose: Treatment

Official Title: A Pharmacokinetic/Pharmacodynamic and Safety Evaluation of Oseltamivir (Tamiflu®) for the Treatment of Children Less Than 24 Months of Age With Confirmed Influenza Infection (CASG 114)

Resource links provided by NLM:

MedlinePlus related topics: Flu Flu Shot
Drug Information available for: Oseltamivir Oseltamivir phosphate
U.S. FDA Resources

Further study details as provided by National Institute of Allergy and Infectious Diseases (NIAID):

Primary Outcome Measures:

- Oseltamivir Carboxylate AUC12 (Area Under the Curve). [Time Frame: Day 3 of drug administration] [Designated as safety issue: No]

  The oseltamivir carboxylate AUC12 was derived from a series of five blood draws over 10 to 12 hours.

Secondary Outcome Measures:

- Overall Reported Adverse Events (AEs) Thought to be Associated With Study Therapy. [Time Frame: Duration of study, from receipt of the first dose of study drug and continuing through study visit Day 30 plus or minus 3 days.] [Designated as safety issue: Yes]

  Any event considered associated with drug that occurred post first dose of drug administration that was not present at baseline was considered an AE. Expected flu symptoms were not reported as AEs but were collected separately.

- Number and Characteristics of Adverse Events (AEs) Described as Neurological Events. [Time Frame: Duration of study, from receipt of the first dose of study drug and continuing through study visit Day 30 plus or minus 3 days.] [Designated as safety issue: Yes]

  Any neurological event that occurred post first dose of drug administration that was not present at baseline was considered an AE. Expected flu symptoms were not reported as AEs but were collected separately.

- Incidence of Treatment Emergent AEs and Drug Related AEs by Cohort and Toxicity Grade [Time Frame: Duration of study, from receipt of the first dose of study drug and continuing through study visit Day 30 plus or minus 3 days] [Designated as safety issue: Yes]

  Any event considered to be related to the study drug that occurred post first dose of drug administration that was not present at baseline was considered an AE. Expected flu symptoms were not reported as AEs but were collected separately. The Division of AIDS Toxicity Tables (DIAIDS) were used to grade the events.

- Incidence of Treatment Emergent AEs and Drug Related AEs by Cohort Leading to Discontinuation of Study Medication [Time Frame: Duration of study, from receipt of the first dose of study drug and continuing through study visit Day 5 plus or minus 1 day] [Designated as safety issue: Yes]

  Study drug was administered for 5 days; any event that occurred prior to the last dose of study medication that was considered related to an AE and that caused the subject to stop taking study drug.

- Incidence of All Serious Adverse Events by Cohort and System Organ Class (SOC) [Time Frame: Duration of study, from receipt of the first dose of study drug and continuing through study visit Day 30 plus or minus 3 days] [Designated as safety issue: Yes]

  Serious Adverse Event (SAE) were classified by MedDRA System Organ Class (SOC). An SAE was reported if it met the following criteria and occurred after the first dose of study medication through the end of the study: death throughout study participation; life threatening; requires inpatient hospitalization or prolongation of existing hospitalization during the period of protocol defined surveillance; results in congenital anomaly or birth defect; results in a persistent or significant disability; and an event considered serious by the PI.

- Correlation of Clearance of Viral RNA by Culture With Pharmacokinetic Parameters by Cohort [Time Frame: Day to negative viral load for subjects positive at baseline] [Designated as safety issue: No]

  The Spearman coefficient and the p-values were computed between the clearance of Viral RNA and Oseltamivir Carboxylate Area under the curve from 0 to 12 hours (AUC0-12)

- Correlation of Clearance of Viral RNA by Polymerase Chain Reaction (PCR) to Pharmacokinetic Parameters by Cohort. [Time Frame: From date of enrollment until the date of first documented absence of viral load by culture, assessed up to 10 days after enrollment.] [Designated as safety issue: No]

  The Spearman coefficient and the p-values were computed between the clearance of viral RNA and oseltamivir carboxylate Area Under the Curve from 0 to 12 hours (AUC 0-12)

Enrollment: 87

Study Start Date: January 2007

Study Completion Date: April 2010

Primary Completion Date: March 2010 (Final data collection date for primary outcome measure)

Arms	Assigned Interventions
Experimental: oseltamivir (Tamiflu®)	Drug: oseltamivir (Tamiflu®)<br>Oseltamivir is supplied as a white powder blend for constitution to a suspension. It is supplied in 100 ml amber glass bottles with 30 grams of powder for oral suspension, a plastic adapter, a plastic oral dispenser and a plastic measuring cup. Initially subjects in Cohort I received oseltamivir 30 mg orally twice daily for 5 days. The DSMB recommended on 05-Aug-2009 that weight based dosing of oseltamivir for subjects subsequently enrolled in Cohort I. Based on pharmacokinetic data available as of that date, the initial weight-based dose to be evaluated for Cohort I is 3.5 mg/kg twice a day. Cohort II and Cohort III will receive oseltamivir at 3.0 mg/kg/dose orally twice daily for 5 days. Cohorts IV and V will receive 3.0 mg/kg/dose orally twice daily for 5 days, this dose may be adjusted.

Detailed Description:
Oseltamivir is approved for prophylaxis and treatment of children 1 year of age and older with influenza. Influenza treatments for children under the age of 1 year are needed because mortality from influenza is high among this age group, even when there are no underlying medical conditions. Oseltamivir is frequently used off-label in children less than 1 year of age, with no data supporting the doses being used. Given the risk of severe or fatal influenza infection in infants, the lack of repeat dose pharmacokinetic (PK) data in children less than 2, the need for treatments in this population of children, and the fact that oseltamivir is being used off-label in this population, the current study will systematically study the PK and safety of oseltamivir in children less than 2 years of age with confirmed influenza to determine the appropriate dose to be used in these age groups. This data will be critical to pediatricians caring for these potentially gravely ill infants. This study is a prospective, age-stratified PK/pharmacodynamic (PD) and safety evaluation of oseltamivir therapy in children less than 24 months of age with confirmed influenza infection. Participants will be stratified by age into the following enrollment scheme at study initiation: 12-23 months (Cohort I), 9-11 months (Cohort II), 6-8 months (Cohort III), 3-5 months (Cohort IV) and 0-2 months (Cohort V). At study onset, Cohort II and III will be enrolled simultaneously. Cohorts IV and V will be enrolled sequentially by decreasing age groups predicated upon the PK and safety data from the preceding cohort. In the event of a public health emergency, the Data Safety Monitoring Board (DSMB) or Food and Drug Administration (FDA) may authorize the following modifications to the proposed enrollment plan: the opening of younger age cohorts without the full dataset from the next higher age cohort, the re-opening of previously closed cohorts to obtain additional data and/or the over-enrollment of any of the 5 cohorts. The oldest cohort (Cohort I) may be enrolled at any time during the study. The primary study objective

is to define the PK of oseltamivir and oseltamivir carboxylate in children with confirmed influenza less than 2 years of age. The oseltamivir dose initially evaluated in Cohort I was the approved dose of 30 mg twice a day (bid). However, the oseltamivir carboxylate area under the curve (AUC)12 values for 5 of the 9 subjects enrolled in Cohort I as of August 5, 2009, were below the lower range utilized for the other cohorts in the study, as was the GM AUC12 for Cohort I as a group [(2589 nanograms per hour per milliliter (ngxh/mL)]. As a consequence, the DSMB recommended on August 5, 2009, that the protocol be amended to utilize weight-based dosing of oseltamivir in subjects subsequently enrolled in Cohort I, and to employ the targeted AUC approach used for Cohorts II-V for this cohort as well. Based upon the PK data available as of that date, the initial weight-based dose to be evaluated for Cohort I is 3.5 mg/kg bid. A dose of oseltamivir 3 mg/kg/dose orally bid for 5 days (10 doses) will be administered to the first 9 subjects in each of Cohorts II-III. Additional subjects may be enrolled if the target AUC12 range is not achieved. The proposed dose for subjects enrolled in Cohorts IV and V will be 3 mg/kg/dose orally bid for 5 days (10 doses), although this dose may be adjusted prior to opening Cohort IV or V based on the dose required to achieve the target oseltamivir carboxylate AUC12 range in the previous cohort.

Eligibility

Ages Eligible for Study:	up to 23 Months (Child)
Genders Eligible for Study:	Both
Accepts Healthy Volunteers:	No

Criteria

Inclusion Criteria:

- Signed informed consent from parent(s) or legal guardian(s).
- Age:

Cohort I: 12 - 23 mo. Cohort II: 9 - 11 mo. Cohort III: 6 - 8 mo. Cohort IV: 3 - 5 mo. Cohort V: 0 - 2 mo.

- Confirmed laboratory diagnosis of influenza by viral culture or rapid influenza diagnostic test within 96 hours prior to study enrollment.
- Duration of influenza symptoms less than or equal to 96 hours.

Exclusion Criteria:

- Concomitant vomiting illness that would preclude ability to take drug.
- Immunocompromised subject (e.g., malignancy, congenital agammaglobulinemia, HIV).
- Documented renal impairment (e.g., polycystic renal disease, nephrectomy, renal transplantation, renal agenesis, dialysis requirement, renal failure, nephrotic syndrome at any time prior to enrollment, current receipt of diuretic therapy).
- Documented hepatic impairment (e.g., congenital hepatitis, biliary atresia, cholelithiasis).
- Gastrointestinal abnormality which might hinder absorption of an oral medication.
- Current receipt of inotropic drugs (e.g., epinephrine, norepinephrine, dopamine, dobutamine).
- History of seizures.
- Documented congenital malformations of the central nervous system defined at birth (e.g., hydranencephaly, prosencephaly, spina bifida).

Contacts and Locations

Choosing to participate in a study is an important personal decision. Talk with your doctor and family members or friends about deciding to join a study. To learn more about this study, you or your doctor may contact the study research staff using the Contacts provided below. For general information, see Learn About Clinical Studies.

Please refer to this study by its ClinicalTrials.gov identifier: NCT00391768

Show 32 Study Locations

Sponsors and Collaborators

National Institute of Allergy and Infectious Diseases (NIAID)

More Information

Publications:

Kimberlin DW, Acosta EP, Prichard MN, Sánchez PJ, Ampofo K, Lang D, Ashouri N, Vanchiere JA, Abzug MJ, Abughali N, Caserta MT, Englund JA, Sood SK, Spigarelli MG, Bradley JS, Lew J, Michaels MG, Wan W, Cloud G, Jester P, Lakeman FD, Whitley RJ; National Institute of Allergy and Infectious Diseases Collaborative Antiviral Study Group. Oseltamivir pharmacokinetics, dosing, and resistance among children aged <2 years with influenza. J Infect Dis. 2013 Mar 1;207(5):709-20. doi: 10.1093/infdis/jis765. Epub 2012 Dec 10.

Responsible Party:	National Institute of Allergy and Infectious Diseases (NIAID)
ClinicalTrials.gov Identifier:	NCT00391768 History of Changes
Other Study ID Numbers:	06-0059 N01AI30025C Roche WP-20749; CASG 114
Study First Received:	October 20, 2006
Results First Received:	March 17, 2011
Last Updated:	April 25, 2013
Health Authority:	United States: Federal Government<br>Canada: Ethics Review Committee<br>United States: Food and Drug Administration<br>United States: Institutional Review Board

Keywords provided by National Institute of Allergy and Infectious Diseases (NIAID):
influenza, oseltamivir, Tamiflu®, antiviral,
children, infants

Additional relevant MeSH terms:
Oseltamivir
Influenza, Human
Orthomyxoviridae Infections
RNA Virus Infections
Virus Diseases
Respiratory Tract Infections
Respiratory Tract Diseases
Antiviral Agents
Anti-Infective Agents
Enzyme Inhibitors
Molecular Mechanisms of Pharmacological Action
