# Supplementary material for: Improving data transparency in clinical trials using blockchain smart contracts
Source: F1000Res. 2016 Oct 20;5:2541. [Version 1] doi: 10.12688/f1000research.9756.1 (PMC5357027; doi:10.12688/f1000research.9756.1)
Supplement: Peer review history

# Peer review: Referee response for version 1

**DOI:** 10.5256/f1000research.10518.r20163

**Published:** 2017-03-16

**Authors:** Jesse Yli-Huumo

**Affiliations:** 1 Department of Computer Science, Aalto University, Helsinki, Finland

This article discusses and proposes a blockchain based smart contract system to improve transparency of clinical trials. The current issues in scientific credibility in clinical trial findings include missing data, endpoint switching, data dredging, and selective publication. There have been efforts to solve these issues with legislation, but it can be seen that they do not solve the issues alone. Prior to this paper, a blockchain method was described to solve these issues. This paper extends the idea by using smart contracts with blockchain.

 The paper is well-written and referenced. I have few questions that need more clarification:

  

1. Regarding FDA regulations, “ *more than half of trials have failed to do so*”. What were the key reasons why this happened? This could be contrasted to issues that this type of blockchain would solve.
2. How is “proof-of-existence” verified by network? How is verification orchestrated in the network? What resources are required from participants to join this blockchain?
3. **Untrusted participants. **Can you elaborate more what do you mean by this term?
4. **EMA. **What is EMA in this picture and does it have some special role compared to other participants?
5. **CDMS. **How is data synchronized and standardized between various participants? What does it require from participant perspective?

Checklist

- The appropriateness of the title: OK
- Whether the abstract provides an adequate summary of the article: OK
- If there is a comprehensive explanation of study design, methods and analysis, and their suitability to the investigation: OK
- Whether the conclusions are balanced and justified on the basis of the results: OK
- With regards to the data (if applicable), whether sufficient information has been provided for replication of the experiment, and/or if the data are in a usable format: OK

Overall, I think this study is really interesting and I enjoyed reading it. Blockchain 2.0 and its applications are now emerging in various fields and it is important that research is being conducted. Looking forward for more results regarding this study in future.

I have read this submission. I believe that I have an appropriate level of expertise to confirm that it is of an acceptable scientific standard.

# Peer review: Referee response for version 1

**DOI:** 10.5256/f1000research.10518.r20706

**Published:** 2017-03-15

**Authors:** Sönke Bartling

**Affiliations:** 1 Department of Medical Physics in Radiation, German Cancer Research Center (DKFZ), Heidelberg, Germany

This article represents the first description of a real-world implementation of blockchain, smart contracts and decentralized/floating cloud storage for medical approval studies.

 It logically extends the application of blockchain beyond the pure ‘notarization’ functionality, as earlier described, into the realm of post-processing and data analyses. The source code is provided, so future groups can work on that.

 It can be hoped that this example will be picked up and used as a model in real-world studies. I ask the author to consider crypto-assured study blinding in future designs. Furthermore, it would be great to compare the practical possibilities of such a study system to today's system on a step-by-step analysis.

 The author mentions the potential of zero-knowledge proofs and homomorphic encryption. I’m looking forward to reading more from this author.

I have read this submission. I believe that I have an appropriate level of expertise to confirm that it is of an acceptable scientific standard.

# Peer review: Referee response for version 1

**DOI:** 10.5256/f1000research.10518.r17773

**Published:** 2016-11-29

**Authors:** William J. Knottenbelt

**Affiliations:** 1 Department of Computing, Imperial College London, London, UK

This article proposes a smart-contract based system for improving the transparency of clinical trials. This represents an original extension to existing proposals which apply blockchain technology to prove the historical existence of certain documents and protocols. Specifically, a private blockchain network with an hierarchical arrangement of smart contracts gives the various stakeholders (e.g. regulators, clinical research organisations etc.) the ability to manage and record trial data in a natural - but also immutable and auditable - fashion.

 The paper itself is well-written and focused and adequately referenced. I have a few minor queries which could be clarified in any final version:

1. "Untrusted parties" - are these actually "untrusting parties" or "untrusted and untrusting" parties?
2. "ensuring tamper resistance" - should this be "ensuring a very high degree of tamper resistance" (since 51% attacks are still possible)? Being a private, permissioned blockchain, does it need any special arrangements to ensure its integrity, or does the fact that runs on top of the Ethereum network ensure this?
3. "includes a container" - can you please elaborate on what you mean by "container" here? It is a very overloaded term in computer science, and can refer to data structures, a lightweight virtual environment for code execution, a file store etc.

Overall I enjoyed reading the paper and I think it makes a worthy contribution to the literature. I am happy to recommend it be indexed.

I have read this submission. I believe that I have an appropriate level of expertise to confirm that it is of an acceptable scientific standard.
